# Trafficking of ‘immune’ CD4^+^/CD8^+^ T-lymphocytes into the RENCA tumour microcirculation in vivo in mice

**DOI:** 10.1054/bjoc.2000.1403

**Published:** 2000-10

**Authors:** S A Ali, R C Rees, D Q Anderson, M W R Reed, J R Goepel, N J Brown

**Affiliations:** Department of Life Sciences, Nottingham Trent University, Clifton Lane, Nottingham, NG11 8NS; Department of Surgical and Anaesthetic Sciences, Pathology, University of Sheffield, Royal Hallamshire Hospital, Glossop Road, Sheffield, S10 2JF

**Keywords:** leucocyte/lymphocyte trafficking, CD4^+^, CD8^+^, tumour microcirculation, immunotherapy

## Abstract

RENCA-IL-2 (Murine Renal Cell Carcinoma transfected with murine IL-2 gene) cells were rejected by immunocompetent (but not T-cell deficient) Balb/c mice, which developed ‘immunity’ to subsequent parental RENCA tumour cell challenge. Splenocytes adoptively transferred this immunity. CD4^+^ and CD8^+^ T-lymphocytes prepared from the spleens of ‘tumour immune’ mice were evaluated for their ability to traffic into the tumour environment using an in vivo model that enables visualization of events within the microvasculature. RENCA cells were implanted into the mouse cremaster muscle and the trafficking of syngeneic lymphocyte subpopulations, derived from naive and ‘immune’ animals, into both the RENCA tumour and the surrounding normal cremaster muscle microcirculation was measured by in vivo microscopy. Fluorescently labelled CD4^+^ and CD8^+^ T lymphocytes taken from the spleens of naive mice or mice previously immunized with RENCA-IL-2 were injected systemically into tumour-bearer mice. Naive effector cells migrated to, and flowed through both the tumour and the normal microcirculation, with negligible adhesion. However we observed the selective recruitment, localization and arrest of immune CD4^+^ and CD8^+^ T lymphocytes (*P* < 0.05) into the tumour microcirculation, and in some instances the subsequent extravasation of cells into the tumour interstitium. Lymphocyte rolling by ‘immune’ CD4^+^ and CD8^+^ T-cells in the tumour microcirculation was greatly reduced, suggesting impaired adhesion molecule expression on the tumour endothelium. This study clearly demonstrates, by direct in vivo microscopy assessment, the localization of effector cells, CD4^+^ and CD8^+^ lymphocytes into tumours. © 2000 Cancer Research Campaign


					Currently unavailable

				

